# Chemical Composition and Antimicrobial Activity of Essential Oils from the Aerial Parts of *Pinus eldarica* Grown in Northwestern Iran

**DOI:** 10.3390/molecules24173203

**Published:** 2019-09-03

**Authors:** Tayyebeh Ghaffari, Hossein Samadi Kafil, Solmaz Asnaashari, Safar Farajnia, Abbas Delazar, Su Cheol Baek, Hamed Hamishehkar, Ki Hyun Kim

**Affiliations:** 1 Drug Applied Research Center, Student Research Committee, Tabriz University of Medical Sciences, Tabriz 51656-65811, Iran; 2 Drug Applied Research Center, Tabriz University of Medical Sciences, Tabriz 51656-65811, Iran; 3 Biotechnology Research Center, Tabriz University of Medical Sciences, Tabriz 51656-65811, Iran; 4 Research Center for Evidence based Medicine, Tabriz University of Medical Sciences, Tabriz 51656-65811, Iran; 5 School of Pharmacy, Sungkyunkwan University, Suwon 16419, Korea

**Keywords:** *Pinus eldarica*, essential oil, chemical profile, antimicrobial activity, β-pinene, β-myrcene, caryophyllene, limonene

## Abstract

*Pinus eldarica* (Pinaceae), an evergreen plant, is distributed across the warm and dry climates of western Asia, including Asia Minor, the Middle East, and land surrounding the Caspian Sea. Essential oils (EOs) from different aerial parts of this tree have been used in traditional medicine. We aimed to investigate the chemical profile and antimicrobial activity of the EO from *P. eldarica* grown in northwestern Iran. EO from the needles, bark, and pollen were extracted with boiling water using a Clevenger apparatus at yield of 0.7–1.2 cm^3^/100 g of dry plant material. The main chemical components of the EO from the needles were D-germacrene (18.17%), caryophyllene (15.42%), γ-terpinene (12.96%), and β-pinene (10.62%); those from the bark were limonene (16.99%), caryophyllene oxide (13.22%), and drimenol (13.2%); and those from the pollen were α-pinene (25.64%) and limonene (19.94%). In total, 83 constituents were characterized in the EOs, using gas chromatography mass spectrometry analysis; mainly, sesquiterpene hydrocarbons in needle EO and monoterpene hydrocarbons in pollen and bark EOs. β-Pinene, β-myrcene, limonene, and caryophyllene were identified in the EOs from all three plant parts. The antibacterial and antifungal properties of the EOs were examined: pollen EO exhibited antibacterial activity against *Escherichia coli*; bark EO inhibited the growth of *Candida albicans* and *Staphylococcus aureus*; and the needle EO inhibited the growth of *S. aureus*. Thus, the EOs from aerial parts of *P. eldarica* can benefit the EO industry and antibiotic development.

## 1. Introduction

*Pinus eldarica*, also known as the Tehran pine, is a coniferous tree of the family Pinaceae. The trees grow to a height of 12–15 m and have a brownish-gray or light gray bark. The needles are paired, medium green, and reach a length of 6–9 cm. The cones may be either solitary or in pairs. *P. eldarica* is native to west Asia and is adapted to warm and dry climates. The trees grow on a wide variety of soil types and have been extensively planted in Iran [1,2]. Pines have been used in medicine and industry throughout human life. Pine needle essential oils (EOs) are used to add odors to products in the soap and perfume manufacturing industry. Previously, the analgesic, anti-inflammatory, and antiseptic effects of pine EOs have been reported [3].

*P. eldarica* has been used for the treatment of asthma [4]; moreover, it is useful in treating eczema and other skin wounds and irritations [5]. In previous studies, it has been shown that the EO from the bark of *P. eldarica* contains polyphenolic compounds, such as taxifolin and catechin, which have a wide range of pharmacological activities [6]. Owing to the important therapeutic applications of *P. eldarica* in traditional medicine, the composition of the EOs from the needles, fruits, and bark of *P. eldarica* grown in Isfahan and Iran has been investigated [2,7]. It was found that D-germacrene, β–caryophyllene, α-pinene, and longifolene were the main components of the EOs of the needles, fruits, and bark of *P. eldarica*, respectively. The composition of resins and needle EOs from *P. sylvestris*, which is native to Europe and Asia, has also been assessed in previous studies [3,8]. The main constituents were δ-3-carene, α-pinene, δ-cadinene, β-pinene, and camphene. The biological activities of the essential EOs of pines have been reported in different parts of the world [9]. The main components of pine extracts, such as α-pinene and β-pinene, have displayed a wide spectrum of antimicrobial activities [10,11]. Previous studies have shown that pine extracts also have potential antifungal properties [12,13].

The composition and content of the EOs of *P. eldarica* may differ depending on the area where these pines are grown. To the best of our knowledge, this is the first study to analyze the EOs from the needles, bark, and pollen of the *P. eldarica* grown in northwestern Iran. The present study was designed to elucidate the chemical profile and antimicrobial activity of these EOs. With growing interest surrounding the use of EOs in the pharmaceutical industry, the results of this study may reveal a new horizon for the development of new pharmaceutical products.

## 2. Results & Discussion

### 2.1. Chemical Composition

EOs were extracted from the aerial parts of *P. eldarica* via hydrodistillation. The yields of the EOs obtained were 1.2 cm^3^/100 g of dried needles, 0.7 cm^3^/100 g of dried pollen, and 0.9 cm^3^/100 g of dried bark (Table 1). These very low yields were consistent with those obtained in previous studies conducted on other species within the genus *Pinus* [14]. Therefore, it seems that this tree is an EO-poor plant.

**Figure 1:**
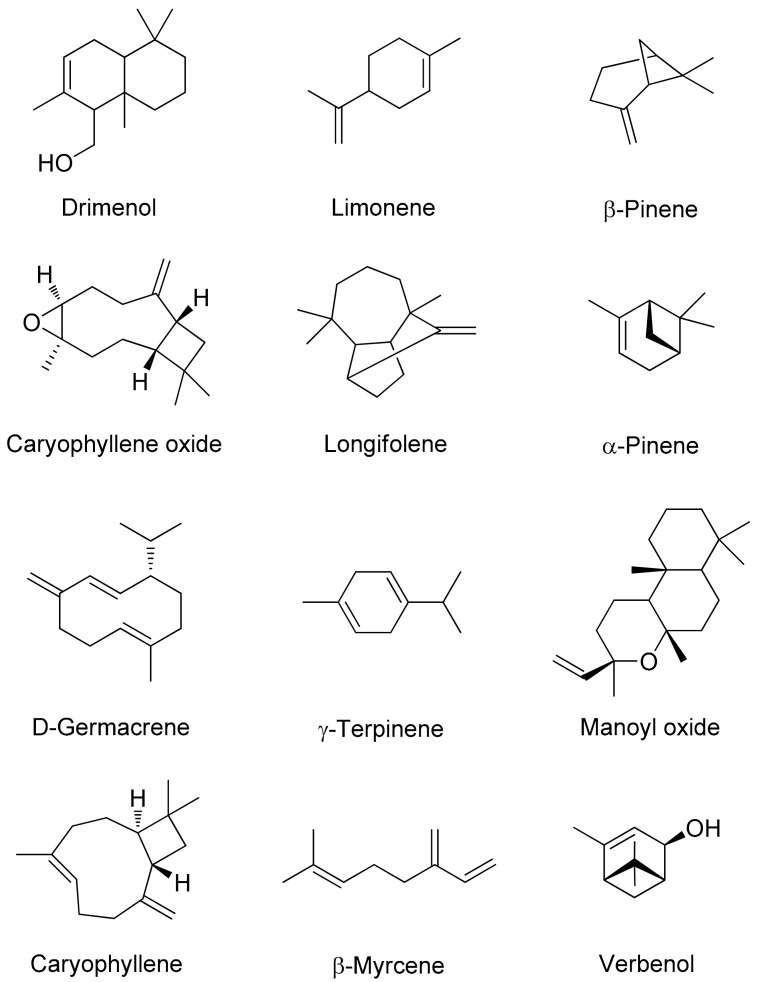
Some chemical components of essential oils obtained from the aerial parts of *Pinus eldarica* using hydrodistillation.

The chemical compositions of the EOs from the needles, bark, and pollen of *P. eldarica* were analyzed using gas chromatography mass spectrometry (GC/MS) and are shown in Table 2 (The Gas chromatogram, condition and method are showed in the Supplementary Materials). The experimental retention indices (RI) of the chemicals in non-polar column and their percentage peak areas are also shown. The differences between the EOs of the needles, bark, and pollen were studied, and the main components of the EOs were identified. Fifty-eight constituents representing 99.98% of the needle EO, 11 components representing 99.94% of the pollen EO, and 33 components representing 99.79% of the bark EO were identified (Table 2). The main fraction of the EO derived from the dried needles was found to consist of sesquiterpene hydrocarbons (49.25% of the EO), which is in agreement with the results of previous studies [2,15]. Monoterpene hydrocarbons (32.91% of the EO) made up the main fraction of the EO derived from dried bark; this was also found in a previously reported study [7]. In the pollen EO, monoterpene hydrocarbons (61.44% of the EO) made up the main fraction, which has not been reported previously. The EOs of the three different aerial parts of *P. eldarica* were mostly composed of hydrocarbon compounds (Figure 1); caryophyllene, limonene, caryophyllene oxide, α-pinene, and β-pinene were identified in all parts.

The main constituents of the needle EO were the sesquiterpene hydrocarbons D-germacrene (18.17%) and caryophyllene (15.42%) and the monoterpene hydrocarbons β-pinene (10.62%) and γ-terpinene (12.96%). The most abundant compound, d-germacrene, has been known to exert cytotoxic activity against cancer cell lines, fungicidal activity, and antibacterial properties against both gram-positive and gram-negative bacteria [16,17]. In a previous study, the main constituents of the *P. eldarica* needle EO were determined to be d-germacrene and β-caryophyllene [15]. This is consistent with the results of our study.

Among the monoterpene hydrocarbons that were found in needle EO, γ-terpinene (12.96%) and β-pinene (10.62%) were the most abundant. It has been reported that γ-terpinene possesses potent antioxidant and anti-inflammatory activity. Treatment with γ-terpinene has been shown to reduce inflammatory parameters, such as edema and cytokine production [18]. In a previous study, α-pinene and β-pinene were determined to be the main compounds in *P. eldarica* [2]. Moreover, α-pinene (0.1–30.8%) and δ-3-carene (1.0–25.5%) were found to be the major components of the Lithuanian *P. sylvestris* needle EO [19]. It can hence be concluded that γ-terpinene has not been found in high amounts in other pines in previous studies. According to previous studies, even EOs from plants of the same species can differ in their composition according to the geographical location and age of the plant [20,21].

**Figure 2:**
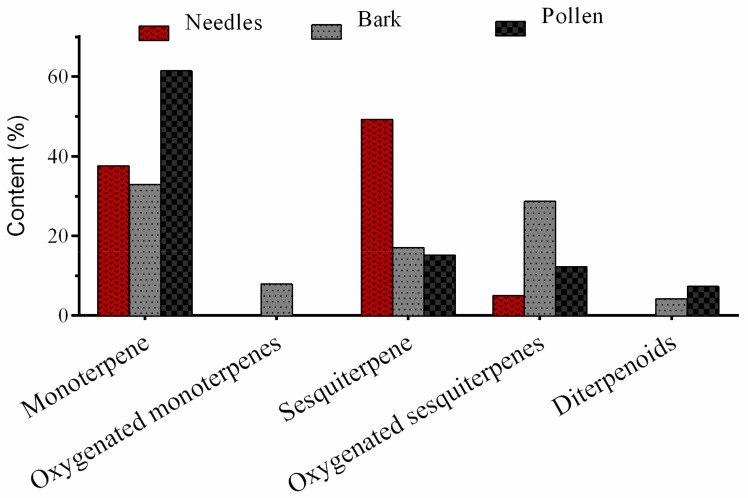
Chemical groups identified in the essential oils obtained from different aerial parts of *Pinus eldarica*.

In the present study, in the pollen EO, α-pinene (25.64%) and limonene (19.94%) were the most abundant compounds (Table 2). Pharmacological studies have suggested that low exposure to α-pinene leads to anti-inflammatory activity via the suppression of mitogen-activated protein kinases (MAPKs) [22]. It has also been shown that α-pinene has potential anti-osteoarthritic [23], antimicrobial [24], antiulcerogenic, and gastroprotective properties [25]. According to the results of the analysis of the bark EO, there was a high proportion of the monoterpene hydrocarbon limonene (16.99%); moreover, oxygenated sesquiterpenes, such as caryophyllene oxide (13.22%) and drimenol (13.2%), were the next most abundant components. Limonene has been reported to possess potent antioxidant and anti-inflammatory properties [26] and inhibit the growth of cancer cells by interfering with the action of G proteins involved in cell signaling pathways [27]. Caryophyllene oxide, an oxygenated terpenoid has been shown to exert significant antifungal [28] and anticancer activities via the suppression of cellular growth and induction of apoptosis [29]. In a previous study of the bark of *P. eldarica* from Isfahan and Iran, α-pinene and caryophyllene oxide were the main constituents of the EO [7]. This result differed from that of the current study, possibly due to difference in the area the studied pines were grown. A classification of the samples based on structure type of EOs obtained from different aerial parts of *P. eldarica* is summarized in Figure 2. A large number of sesquiterpene hydrocarbons (49.25%), monoterpene hydrocarbons (37.57%), and oxygenated sesquiterpenes (5.05%) were detected in the needle EO in this study, whereas the content of diterpenoids was significantly lower (0.23%). The pollen and bark EO had a large abundance of monoterpene hydrocarbons (61.44% and 32.91%, respectively). Considering all these data together has shown that there is quantitative variation between species and between different aerial parts of *Pinus*. This information could be valuable for the chemotaxonomic study of *Pinus* species.

### 2.2. Antimicrobial Activity

Recently, the antibiotic-resistant bacterial pathogens are increasing and resulting in a reduction in the efficiency of clinical treatments. *Staphylococcus aureus* and *Escherichia coli* have a high ability to become resistant to antibiotics. Latest reports of vancomycin-resistant *S. aureus* have shown that effective antibiotics against the organism may not be readily available for a long time [30]. It was demonstrated that 71.6% of *E. coli* strains isolated from children’s stool and water samples in South Africa were multi-antibiotic resistant [31]. Several studies have focused on natural plant products with potent anticancer, antibacterial, antifungal, and antioxidant effects, and the efficacy of EOs have been emphasized [32,33,34]. According to our study, the EOs of *P. eldarica* contain monoterpene hydrocarbons, sesquiterpene hydrocarbons, and oxygenated hydrocarbons. In this study, the antibacterial and antifungal properties of the EOs of *P. eldarica* were examined. The results of the antimicrobial assay are presented in Table 3. Both needle and bark EOs displayed antibacterial activity against *S. aureus* (ATCC 29213) and pollen EO exhibited a bactericidal effect against *E. coli* (ATCC 25922). *Candida albicans* (ATCC 10231) was most sensitive to the bark EO, with a minimum inhibitory concentration (MIC) value of 125 µg/mL. These EOs of *P. eldarica* contain major compounds, such as α-pinene, germacrene, caryophyllene, and limonene, which have been reported to display antimicrobial activity against important pathogens. Rivas da Silva et al. [35] tested the biological activities of α-pinene and β-pinene enantiomers against *C. albicans*, *Cryptococcus neoformans* T_1_-444 Serotype A, *Rhizopus oryzae* UCP1506, and *S. aureus* using the MIC and the minimal microbicidal concentration (MMC). They reported that the (+)-enantiomers showed high antifungal activity and that the synergistic effects of these compounds combined with microbicides reduced the MIC of the combined materials [35]. In the similar study performed by Scalas et al. [36], the EO of *P. sylvestris* and α-pinene displayed good inhibitory activities against *C. neoformans*. In addition, combination of itraconazole with the EO of *P. sylvestris* showed a good synergistic action against *C. neoformans*. The EO of *Liquidambar styraciflua* leaf, which includes α-pinene as its major compound, also showed a good synergistic effect with tetracycline and ciprofloxacin against *Bacillus subtilis* (ATCC 6633) [37]. In the present study, EO of pollen was rich in α-pinene (25.64%) and exhibited moderate antibacterial activity against *E. coli* with MIC level of 225 μg/mL. Recently, one study reported the moderate antifungal activity of *Thimus algeriensis* EO against *C. glabrata* (ATCC22553) and *C. albicans* and a total of 29 compounds were identified in the EO of *T. algeriensis*, with α-terpinyl acetate (47.4%), neryl acetate (9.6%), and α-pinene (6.8%) as the major compounds [38]. Moreover, the antimicrobial activity of *Juniperus oxycedrus* EO was found against *S. aureus* subs. *aureus* (CCM 4223) and predominant constituent of *J. oxycedrus* EO was also identified as α-pinene [39]. In a study of the antifungal activity of *P. pinaster* bark, its ethanolic extract displayed high antifungal activity against *Trametes versicolor* and moderate antifungal activity against *Coniophera puteana* [40]. It was shown that limonene has antibacterial activities against *S. aureus* (gram-positive), *Pseudomonas aeruginosa* (gram-negative), and the yeast, *C. neoformans* [41]. The antibacterial activities of *P. elliottii* resin-oil against multidrug-resistant strains were evaluated using the minimum inhibitory concentration (MIC) method. The MIC varied between 25 and 100 µg/mL, and the drug-resistant mutant of *Staphylococcus epidermidis* was more sensitive to the oils [42].

## 3. Materials and Methods

### 3.1. Plant Material

Pine needles, bark, and pollen were collected from the Tabriz district in Iran. The samples were collected in June 2018 and identified (No. 4036) by the Herbarium of the School of Pharmacy, Tabriz University of Medical Sciences. The specimens were air dried at room temperature, powdered, and stored in airtight bottles at 4 °C.

### 3.2. Essential Oil Preparation

*P. eldarica* bark, needles, and pollen powders (100 g) were hydro-distilled (with 1.2 L of water) in a Clevenger-type apparatus by recirculating the condensed water. The distillation was terminated after 240 min. The resulting EOs were dissolved in diethyl ether, collected, and treated with anhydrous sodium sulfate to remove excess water. The diethyl ether was removed carefully at room temperature, and the remaining EOs were stored in sealed vials at 4 °C until analysis.

### 3.3. Gas Chromatography Mass Spectrometry (GC/MS) Analysis of the Essential Oils

Analysis of the EOs was carried out using a Shimadzu QP5050A GC/MS instrument (Shimadzu, Kyoto, Japan) at the following conditions: the volume of sample injected was 1 μL; the helium carrier gas flow rate was 1.3 mL/min, with a split ratio 1:8; the injection site temperature was 270 °C; a DB-1 capillary column (60 m × 0.25 mm) was used, with a film thickness of 0.25 μm; the column temperature was 50 °C increasing at 4 °C/min to 300 °C; an ionization potential of 70 eV was used; the source temperature was 300°C; and the mass range was 30–600 m/z.

### 3.4. Identification of Components

The identification of the components was conducted using computer matching against library spectra (Library Database Wiley 229, NIST 21, NIST 107), obtaining their retention indices with reference to an n-alkane series (C8-C20) in a temperature-programmed run, interpreting their fragmentation pattern, and comparing the mass spectra with relevant reference samples and the literature [43,44].

### 3.5. Microbial Strains and Culture Media

All microbial strains were obtained from the Microbiology Laboratory, Drug Applied Research Center, Tabriz University (Tabriz, Iran) of Medical Sciences. Stock cultures of gram-positive *S. aureus* (ATCC 29213), gram-negative *E. coli* (ATCC 25922), and *C. albicans* (ATCC 10231) were sub-cultured and maintained on nutrient agar at 37 °C for 24 h; subsequently, they were diluted in sterile saline solution (0.85% *w/v*) to reach a final concentration of 0.5 McFarland (~1.5 × 10^8^ colony-forming units [CFUs]/mL).

### 3.6. Determination of the Minimum Inhibitory Concentration

Briefly, the EOs were dissolved in dimethyl sulfoxide and 180 µL was serially diluted from 2000 µg/mL to 10 µg/mL (up to eight dilutions) in Mueller-Hinton broth (Merck KGaA, Darmstadt, Germany). These dilutions were added to 96-well microplates, and 20 µL of the microbial cultures were added at a concentration of 1.5 × 10^8^ CFU/mL to reach a final volume of 200 µL/well. Gentamicin was used as the positive control as it is a broad-spectrum antibiotic, and normal saline/dimethyl sulfoxide (DMSO) was used as the negative control. To complete the test, each organism was suspended separately in 200 µL of Mueller–Hinton broth. *C. albicans* (ATCC 10231) was cultured in Mueller–Hinton agar supplemented with 1% glucose. All the tests were performed in triplicate to ensure reliability of the results. The sealed microplates were mixed on a plate shaker at 300 rpm for 30 s. They were then incubated at 37 °C for 24 h and observed for growth or turbidity; subsequently, the MIC was determined. The MIC was defined as the lowest concentration of the EO that inhibited the visible growth of a microorganism after overnight incubation [45]. For each well showing no growth, a loopful of broth was then sub-cultured on a nutrient agar plate (Merck KGaA, Darmstadt, Germany) to verify if the growth of the microorganism had been inhibited. The growth of the microorganism on the solid media indicated that the specific concentration of the EO was unable to inhibit the growth of the microorganism.

## 4. Conclusions

This is the first study, to our knowledge, that investigated the chemical composition and antimicrobial activity of EOs from the aerial parts of *P. eldarica* grown in northwestern Iran. The main components of the EOs were sesquiterpene hydrocarbons in the needles and monoterpene hydrocarbons in the pollen and bark. In particular, β-pinene, β-myrcene, limonene, and caryophyllene were the most abundant chemicals identified in the EOs of all three plant parts. The EOs demonstrated antimicrobial activity against some highly susceptible strains, *E. coli, C. albicans*, and *S. aureus.* The results of the study provide experimental evidence that EOs from aerial parts of *P. eldarica* can be useful in the EO industry and in the development of antibiotics. In addition, *P. eldarica*, which is widely grown in Iran, can be used to provide low-cost therapies in land surrounding the Caspian Sea.

## Figures and Tables

**Table 1:** Yield of essential oils from aerial parts of *Pinus eldarica.*

Plant Materials	Yield of EO *
Needles	1.2
Pollen	0.7
Bark	0.9

* (cm^3^/100 g of dry plant material).

**Table 2:** The results of gas chromatography mass spectrometry (GC/MS) analysis of the chemical composition of essential oils obtained from the aerial parts of *Pinus eldarica* using hydrodistillation.

		Needles	Bark	Pollen	
No.	Component ^a^	Content, %			RI ^b^
1	n-Hentriacontane	0.59	-	-	472
2	α-Pinene	t	-	25.64	928
3	Camphene	2.95	1.87	-	940
4	Verbenene	-	1	-	949
5	β-Pinene	10.62	2.82	3.12	967
6	β-Myrcene	2.85	1.35	4.34	981
7	α-Phellandrene	t	-	-	994
8	Carene	0.12	-	2.98	1017
9	β-Phellandrene	0.2	-	-	1019
10	Limonene	4.79	16.99	19.94	1020
11	Cis-Ocimene	0.11	-	-	1025
12	β-Ocimene	2.07	-	-	1036
13	γ-Terpinene	12.96	-	-	1047
14	α-Terpinolene	0.23	-	-	1063
15	α-Pinene oxide	-	0.62	-	1065
16	Linalool	t	0.85	-	1082
17	α-Campholenal	t	-	-	1097
18	α-Campholene aldehyde	-	1.72	-	1119
19	Verbenol	-	2.46	5.42	1132
20	Pinocarvone	-	1.15	-	1145
21	Myrtenal	-	1.65	-	1166
22	α-Phellandren-8-ol	-	2.22	-	1167
23	Myrtenol	-	1.71	-	1182
24	T-Carveol	-	1.28	-	1204
25	Carvone	-	1.17	-	1220
26	2-Cyclopropylidene-1,7,7-trimethylbicyclo[2.2.1]heptane	0.15	-	-	1254
27	Bornyl acetate	3.09	-	-	1264
28	Thymol	t	-	-	1270
29	α-Terpinyl acetate	1.68	-	-	1338
30	Neryl acetate	0.16	-	-	1343
31	α-Cubebene	0.25	-	-	1360
32	α-Copaene	0.49	-	-	1387
33	Longifolene	-	3.44	11.66	1393
34	β-Elemene	0.38	-	-	1398
35	β-Cubebene	0.82	-	-	1420
36	Caryophyllene	15.42	9.43	3.52	1426
37	Aromadendrene	0.23	-	-	1440
38	α-Caryophyllene	3.36	-	-	1454
39	α-Humulene	-	2.26	-	1463
40	α–Amorphene	0.81	-	-	1466
41	D-Germacrene	18.17	0.93	-	1473
42	Valencene	0.51	-	-	1486
43	Tridecanal	0.23	-	-	1489
44	γ –Muurolene	2.07	-	-	1501
45	α–Cadinene	0.13	-	-	1522
46	δ–Cadinene	2.42	-	-	1522
47	γ–Cadinene	0.93	-	-	1525
48	α–Muurolene	0.73	-	-	1537
49	Epiglobulol	0.16	-	-	1548
50	Lauric acid	0.34	-	-	1552
51	Isopatchoulane	0.15	-	-	1552
52	Spathulenol	0.91	-	-	1570
53	4(14)-Salvialen-1-one	0.34	-	-	1580
54	Cedrol	0.33	-	-	1585
55	Caryophyllene oxide	1.03	13.22	12.21	1595
56	Humulane-1,6-dien-3-ol	-	0.88	-	1619
57	γ–Eudesmol	-	0.62	-	1624
58	α–Cadinol	0.51	-	-	1630
59	Viridiflorol	0.25	-	-	1636
60	Cadalene	0.36	-	-	1641
61	Widdrene	0.15	-	-	1644
62	Aromadendrene oxide	0.63	-	-	1650
63	Pinocarveol	-	2.82	-	1654
64	Isospathulenol	0.12	-	-	1667
65	Tumerone	1.76	-	-	1680
66	6-Isopropenyl-4,8a-dimethyl-1,2,3,5,6,7,8,8a-octahydro-naphthalen-2-ol	0.61	-	-	1690
67	1,5-Epoxysalvial-4(14)-ene	0.27	-	-	1945
68	18-Norabieta-8,11,13-triene	-	0.73	-	1969
69	Phenethyl isovalerate	-	0.71	-	1986
70	Palmitinic acid	0.97	-	-	2001
71	Humulene oxide	-	1.61	-	2038
72	13(16),14-Labdien-8-ol	-	1.51	-	2120
73	Hexahydrofarnesyl acetone	0.45	1.6	-	2131
74	Linalyl anthranilate	0.4	-	-	2157
75	Manoyl oxide	-	1.37	7.32	2376
76	Drimenol	-	13.2	-	2494
77	α-Campholenic aldehyde	t		-	-
78	Pinocarveylacetate	0.18		-	-
79	3-Ethyl-3-hydroxyandrostan-17-one	0.23		-	-
80	tricyclo[4.3.0.07,9]nonane,2,2,5,5,8,8-hexamethyl	-	1.02	-	-
81	Acetic acid, bornyl ester	-	4.96	-	-
82	Isopimaric acid	-	0.62	-	-
83	Acetic acid, 1,7,7-trimethyl-bicyclo[2.2.1]hept-2-yl ester	-	-	3.79	-
	Total	99.98	99.79	99.94	
	Monoterpene hydrocarbons	37.572	32.91	61.44	
	Oxygenated monoterpenes	0.22	7.92		
	Sesquiterpene hydrocarbons	49.25	17.08	15.18	
	Oxygenated sesquiterpenes	5.05	28.65	12.21	
	Diterpenoids	0.23	4.23	7.32	

^a^ Compounds listed in order of elution from a DB-1 column; ^b^ RI-retention index on non-polar column; t = trace (<0.1%).

**Table 3:** The minimum inhibitory concentrations (MICs) of essential oils from different aerial parts of *Pinus eldarica* against three microorganisms using gentamicin as a positive control.

Microorganisms	MIC * (µg/mL)			
	Needles	Pollen	Bark	Gentamicin
*C. albicans*	na	1000	125	16
*E. coli*	na	225	na	16
*S. aureus*	125	na	125	16

na—no activity.

## Supplementary Materials

Gas chromatograms from the GC/MS analysis are available free of charge on the Internet.
