# Supplementary material for: Chemical Composition and Antimicrobial Activity of Essential Oils from the Aerial Parts of Pinus eldarica Grown in Northwestern Iran
Source: Molecules. 2019 Sep 3;24(17):3203. doi: 10.3390/molecules24173203 (PMC6749391; doi:10.3390/molecules24173203)
Supplement: Supplementary file 1 [file molecules-24-03203-s001.pdf]

Supporting Information

Chemical Composition and Antimicrobial Activity of Essential Oils from the Aerial Parts of *Pinus eldarica* Grown in Northwestern Iran

Taiyebe Ghaffari [1], Hossein Samadi Kafil [2], Solmaz Asnaashari [3], Safar Farajnia [2], Abbas Delazar [4], Su Cheol Baek [5], Hamed Hamishehkar [2,*] and Ki Hyun Kim [5,*]

[1] Drug Applied Research Center, Student Research Committee, Tabriz University of Medical Sciences, Tabriz, Iran; ghaffari.t@tbzmed.ac.ir (T.G.)
[2] Drug Applied Research Center, Tabriz University of Medical Sciences, Tabriz, Iran; kafilhs@tbzmed.ac.ir (H.S.K.); farajnia@gmail.com (S.F.)
[3] Biotechnology Research Center, Tabriz University of Medical Sciences, Tabriz, Iran; asnaasharisolmaz@gmail.com (S.A.)
[4] Research Center for Evidence based Medicine, Tabriz University of Medical Sciences, Tabriz, Iran; delazara@tbzmed.ac.ir (A.D.)
[5] School of Pharmacy, Sungkyunkwan University, Suwon 16419, Republic of Korea; schii513@daum.net (S.C.B.)

*Correspondence: hamishehkarh@tbzmed.ac.ir (H.H.); khkim83@skku.edu (K.H.K.); Tel.: +98-41-3363311 (H.H.); +82-31-290-7700 (K.H.K.)

1) Gas chromatogram from the GC/MS analysis of the pollen EOs

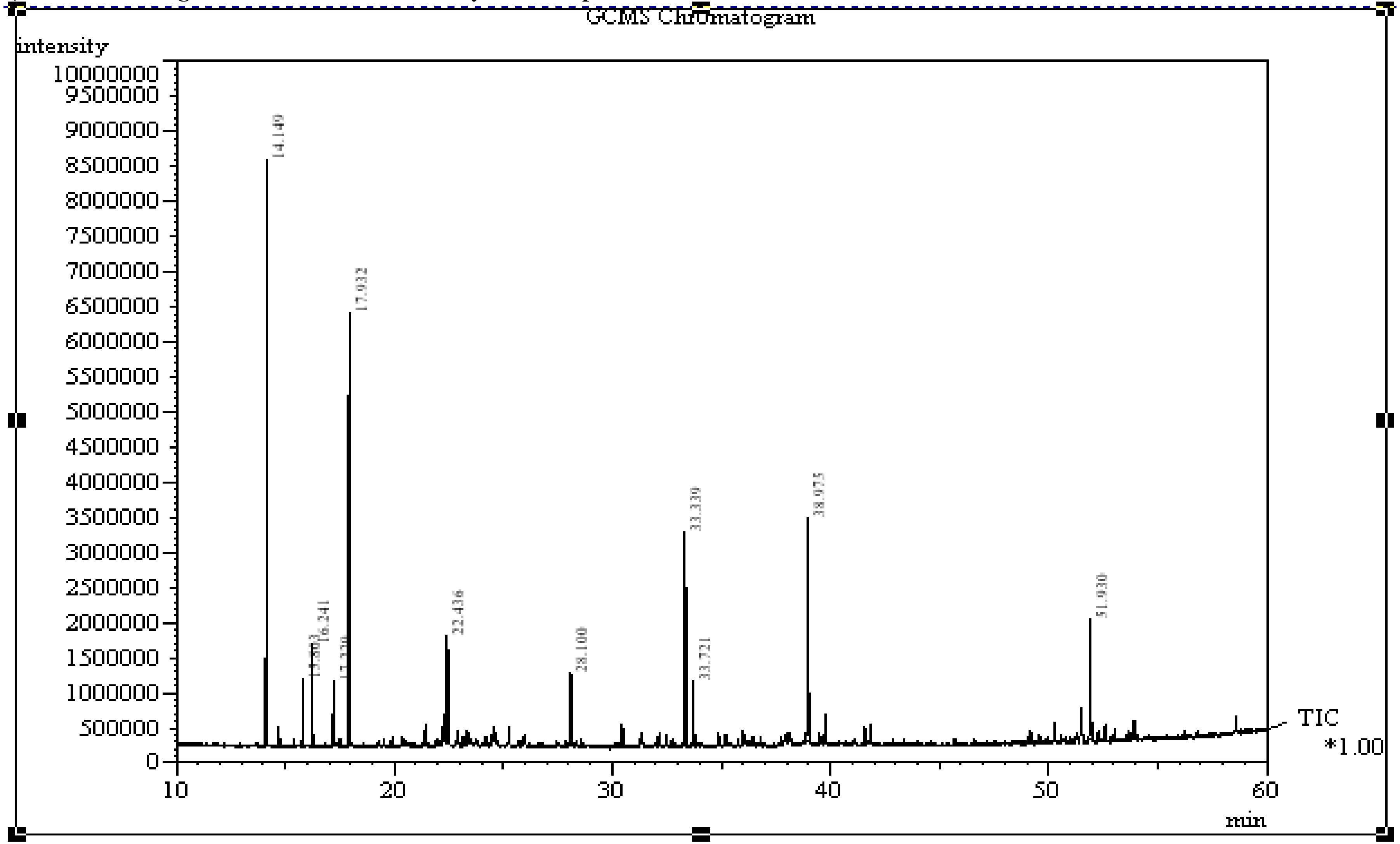

1-1) Condition and method

[GC-17A Ver.3]

Inj. Initial Temp.	:270.00 °C	
Interface Temp.	:300.00 °C	
Control Mode	:Split	
Column Inlet Pressure	:150.0 kPa	
Column Flow	:1.3 mL/min	
Linear Velocity	:29.6 cm/sec	
Split Ratio	:8	
Total Flow	:17.4 mL/min	
Ready Check[COL(Oven)]		:ON
Ready Check[INJ]	:ON	
Ready Check[DET(Interface)]		:ON
Ready Check[AUX1]	:OFF	
Ready Check[AUX2]	:OFF	
Ready Check[AUX3]	:OFF	
Ready Check[AUX4]	:OFF	
Ready Check[AUX5]	:OFF	
Ready Check[Carrier Gus Flow(SPL)]		:ON
Ready Check[Wait]	:OFF	
Equilibrium Time	:1.00 min	
Oven Temp. Program	:	
Rate	Temperature(°C)	Keep Time(min)
-	50.0	2.00
4.0	300.0	6.00
Pressure Program	:	
Rate	Pressure(kPa)	Keep Time(min)
-	150.0	0.00
AUX1 Program	:	
Rate	Temperature(°C)	Keep Time(min)
-	0.0	0.00

[GC Program]

[GCMS-QP5050]

Acquisition Mode	:Scan
Interface Temp.	:300.00 °C
Solvent Cut Time	:2.00 min
Detector Gain Mode	:Relative
Detector Gain	:0.00 kV
Threshold	:1000
Sampling Rate	:0.50 sec

[Scan Group]

No.	Start Time(min)	End Time(min)	Start m/z	End m/z	Scan Speed
1	5.00	70.00	30.00	600.00	2000

Sample Inlet Unit :GC

[MS Program]

Use MS Program :OFF

2) Gas chromatogram from the GC/MS analysis of the bark EOs

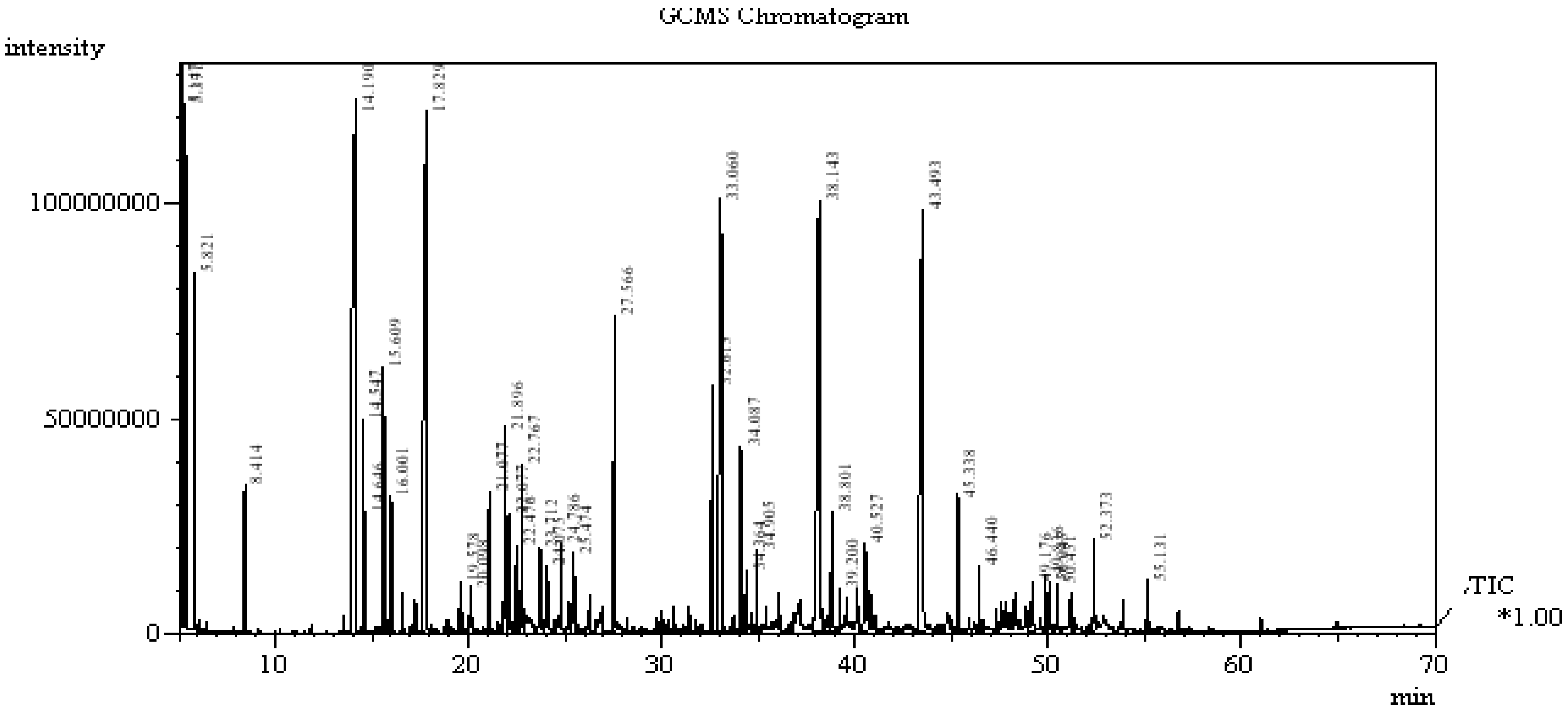

2-1) Condition and method

GCMS Method

[Comment]

[GC-17A Ver.3]

Inj. Initial Temp.	:270.00 °C	
Interface Temp.	:300.00 °C	
Control Mode	:Split	
Column Inlet Pressure	:150.0 kPa	
Column Flow	:1.3 mL/min	
Linear Velocity	:29.6 cm/sec	
Split Ratio	:8	
Total Flow	:17.4 mL/min	
Ready Check[COL(Oven)]		:ON
Ready Check[INJ]	:ON	
Ready Check[DET(Interface)]		:ON
Ready Check[AUX1]	:OFF	
Ready Check[AUX2]	:OFF	
Ready Check[AUX3]	:OFF	
Ready Check[AUX4]	:OFF	
Ready Check[AUX5]	:OFF	
Ready Check[Carrier Gas Flow(SPL)]		:ON
Ready Check[Wait]	:OFF	
Equilibrium Time	:1.00 min	
Oven Temp. Program	:	
Rate	Temperature(°C)	Keep Time(min)
-	50.0	2.00
4.0	300.0	6.00

Pressure Program	:	
Rate	Pressure(kPa)	Keep Time(min)
-	150.0	0.00

AUX1 Program	:	
Rate	Temperature(°C)	Keep Time(min)
-	0.0	0.00

[GC Program]

[GCMS-QP5050]

Acquisition Mode	:Scan
Interface Temp.	:300.00 °C
Solvent Cut Time	:2.00 min
Detector Gain Mode	:Relative
Detector Gain	:0.00 kV
Threshold	:1000
Sampling Rate	:0.50 sec

[Scan Group]

No.	Start Time(min)	End Time(min)	Start m/z	End m/z	Scan Speed
1	5.00	70.00	30.00	600.00	2000

Sample Inlet Unit :GC

[MS Program]

Use MS Program :OFF

3) Gas chromatogram from the GC/MS analysis of the needle EOs

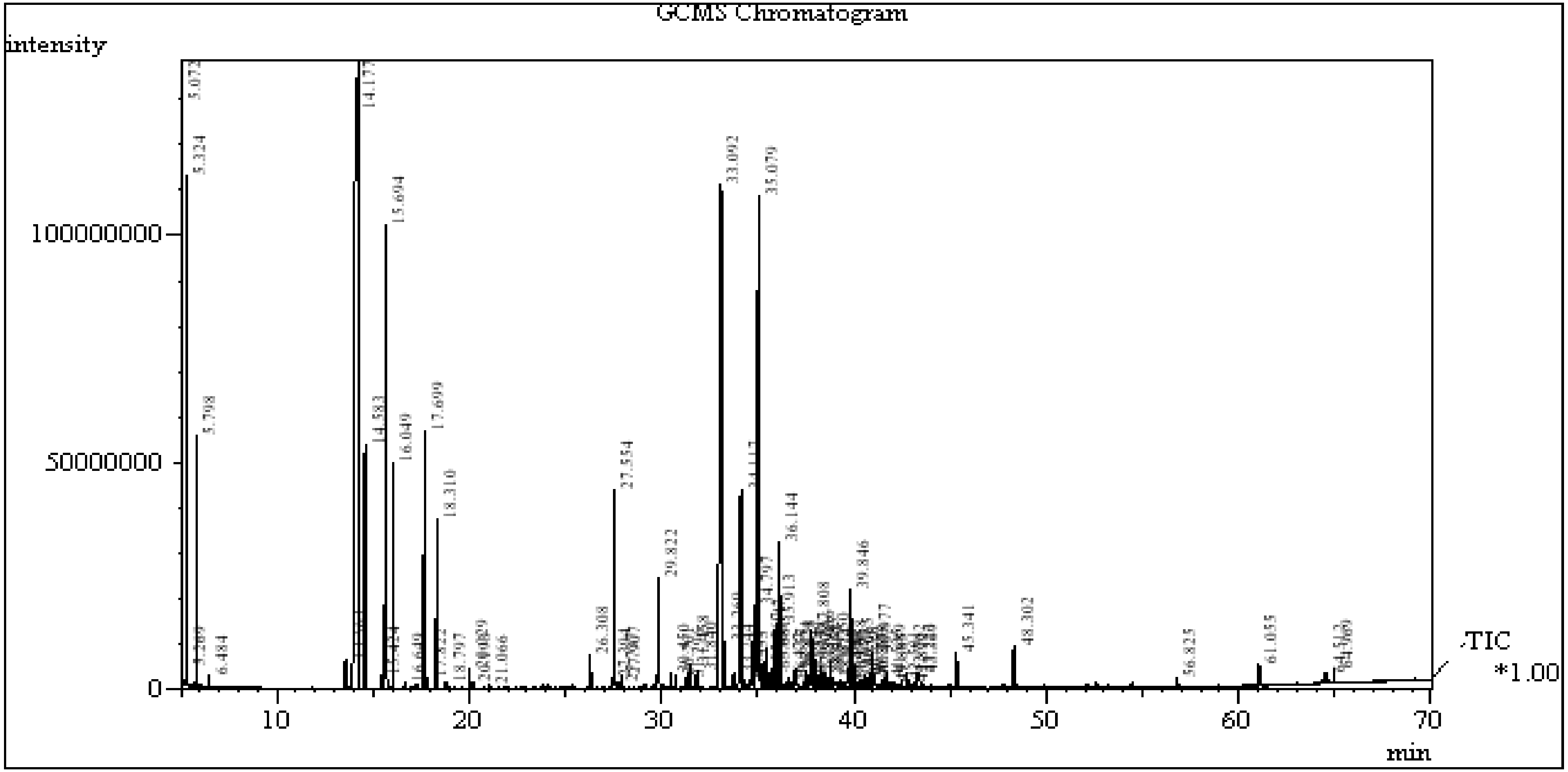

3-1) Condition and method

GCMS Method

[Comment]

[GC-17A Ver.3]

Inj. Initial Temp.	:270.00 °C	
Interface Temp.	:300.00 °C	
Control Mode	:Split	
Column Inlet Pressure	:150.0 kPa	
Column Flow	:1.3 mL/min	
Linear Velocity	:29.6 cm/sec	
Split Ratio	:8	
Total Flow	:17.4 mL/min	
Ready Check[COL(Oven)]		:ON
Ready Check[INJ]	:ON	
Ready Check[DET(Interface)]		:ON
Ready Check[AUX1]	:OFF	
Ready Check[AUX2]	:OFF	
Ready Check[AUX3]	:OFF	
Ready Check[AUX4]	:OFF	
Ready Check[AUX5]	:OFF	
Ready Check[Carrier Gas Flow(SPL)]		:ON
Ready Check[Wait]	:OFF	
Equilibrium Time	:1.00 min	
Oven Temp. Program	:	
Rate	Temperature(°C)	Keep Time(min)
-	50.0	2.00
4.0	300.0	6.00
Pressure Program	:	
Rate	Pressure(kPa)	Keep Time(min)
-	150.0	0.00
AUX1 Program	:	
Rate	Temperature(°C)	Keep Time(min)
-	0.0	0.00

[GC Program]

[GCMS-QP5050]

Acquisition Mode	:Scan
Interface Temp.	:300.00 °C
Solvent Cut Time	:2.00 min
Detector Gain Mode	:Relative
Detector Gain	:0.00 kV
Threshold	:1000
Sampling Rate	:0.50 sec

[Scan Group]

No.	Start Time(min)	End Time(min)	Start m/z	End m/z	Scan Speed
1	5.00	70.00	30.00	600.00	2000

Sample Inlet Unit :GC

[MS Program]

Use MS Program :OFF
